# UKAEA capabilities to address the challenges on the path to delivering fusion power

**DOI:** 10.1098/rsta.2017.0436

**Published:** 2019-02-04

**Authors:** I. T. Chapman, A. W. Morris

**Affiliations:** United Kingdom Atomic Energy Authority, Culham Centre for Fusion Energy, Culham Science Centre, Abingdon, Oxon OX14 3DB, UK

**Keywords:** tokamak, materials, tritium, robotics

## Abstract

Fusion power could be one of very few sustainable options to replace fossil fuels as the world's primary energy source. Fusion offers the potential of predictable, safe power with no carbon emissions and fuel sources lasting for millions of years. However, it is notoriously difficult to achieve in a controlled, steady-state fashion. The most promising path is via magnetic confinement in a device called a tokamak. A magnetic confinement fusion (MCF) power plant requires many different science, technology and engineering challenges to be met simultaneously. This requires an integrated approach from the outset; advances are needed in individual areas but these only bring fusion electricity closer if the other challenges are resolved in harmony. The UK Atomic Energy Authority (UKAEA) has developed a wide range of skills to address many of the challenges and hosts the JET device, presently the only MCF facility capable of operating with both the fusion fuels, deuterium and tritium. Recently, several major new UKAEA facilities have been funded and some have started operation, notably a new spherical tokamak (MAST Upgrade), a major robotics facility (RACE), and a materials research facility (MRF). Most recently, work has started on Hydrogen-3 Advanced Technology (H3AT) for tritium technology and a group of Fusion Technology Facilities.

This article is part of a discussion meeting issue ‘Fusion energy using tokamaks: can development be accelerated?’

## 1. Introduction

Fusion power could be one of a very few sustainable options to replace fossil fuels as the world's primary energy source. Fusion offers the potential of predictable power generators that have no carbon emissions, fuel sources lasting for millions of years and many natural safety features. Fusion is low in land-use, has high energy yield and suitably designed power plants can have very little long-lived radioactive waste and no proliferation issues. In short, it is a highly attractive energy source. However, fusion is notoriously difficult to achieve in a controlled, steady-state fashion on Earth.

The fusion power comes from reactions between two light nuclei. The easiest reaction to initiate is between deuterium and tritium: d + t → ^4^He (3.5 MeV) + *n* (14.1 MeV), where the neutron takes energy to the outside world. The fusion yield becomes significant in plasmas with temperatures in the 10–20 keV range (100–200 million kelvin). These plasmas need to be confined, kept hot and be sufficiently dense to provide fusion power densities on the order of MW m^−3^. The alpha-particles provide most of the heating and the most promising confinement path is via magnetic confinement fusion (MCF), the JET [1] and ITER [2] ‘tokamaks’^1^ being the pre-eminent examples of this approach. Although the conditions for sufficient fusion power density have been reached [3,4] much remains to be done to turn scientific success into commercial electrical power.

An MCF power plant requires many diverse interconnected systems and many different science, technology and engineering challenges to be met simultaneously. ITER and the coordinated European effort designing its successor, DEMO [5,6], have shown that this requires an integrated approach from the outset; advances are needed in individual areas but only bring fusion electricity closer if the other challenges are resolved in harmony. A global systems engineering approach will be used, all the way from the plasma to the turbines, via the blanket—a thermodynamically efficient neutron-to-heat convertor made from materials resilient to neutron damage. All must be buildable, highly reliable and maintainable, mostly robotically, and then endorsed by nuclear regulators and industrial and other stakeholders. This calls for a broad and comprehensive R&D programme combined with innovation and industrial techniques. The UK Atomic Energy Authority (UKAEA) (https://www.gov.uk/government/organisations/uk-atomic-energy-authority) has a mission to make major contributions to the development of fusion power as a large-scale carbon-free commercial energy source. It is and will continue to be a major player in the global fusion enterprise, building on its long involvement in plasma research together with experience of operating JET, constructing the new MAST Upgrade device [7] (http://www.ccfe.ac.uk/mast_upgrade_project.aspx, http://www.ccfe.ac.uk/assets/documents/other/MAST-U_RP_v4.0.pdf) and the more recent expansion into materials science and now wider fusion technology. UKAEA contributes in many areas of science and technology, has growing ties with many universities and increasingly acts as a link to industry, which will be a major contributor and stakeholder in the future. It acts as the hub of UK fusion research and a gateway to the wider communities. This paper focuses on the existing and imminent facilities at Culham and the ways in which they can be exploited by UK, other European and international researchers to address several of the key challenges.

## 2. Challenges on the path to delivering fusion power

**Figure 1.**
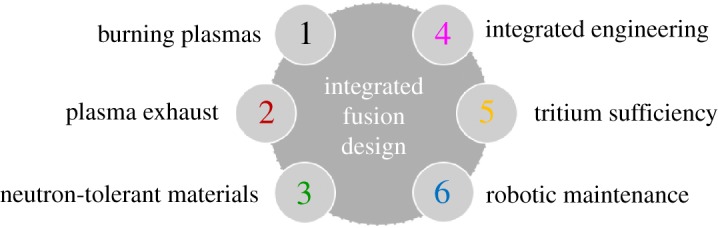
The main challenges which must be overcome to produce an integrated fusion reactor design. The UKAEA portfolio of capabilities seeks to address each of these challenges. (Online version in colour.)

Fusion power relies on the design of integrated solutions for DEMO and power plants, constrained by major technical challenges. This integrated design must simultaneously achieve: (i) the creation and sustainment of a controlled burning plasma over long timescales with fusion-born alpha-particles dominating the plasma heating; (ii) the controlled exhaust of heat and helium ‘ash’ from the burning plasma core; (iii) the development of (a) structural materials for the tokamak structures which have to sustain, for many years, large forces and pressures at high temperatures in the presence of high magnetic fields and exceptionally intense neutron fluxes, without generating unmanageable radioactive waste, and (b) functional materials resilient to neutron and gamma irradiation, e.g. for electrical and thermal insulators, tritium permeation barriers, diagnostic windows and breeding (e.g. lithium-containing ceramics); (iv) the development and design of components with these materials, notably the breeding blanket and plasma-facing components, which can survive in the demanding conditions within a fusion reactor; (v) the requisite high availability and efficiency of the machine and its systems to produce a viable cost of electricity; and (vi) the ability to breed and handle tritium fuel as well as de-tritiate components at end-of-life to minimize tritiated waste. These challenges are depicted in figure 1. These and other constituent parts such as the high field magnets, plasma and plant control systems, buildings and the systems to convert fusion power to electricity must be brought together in an integrated multi-disciplinary nuclear design satisfying regulation and safety requirements. Fusion is different from most other technologies in that a full test is only possible in a complete device, and the cost and timescale of each step means that a succession of small-increment full physical prototypes is unrealistic. Making large steps leads to two additional challenges: (vii) development of extensive theory-based models and an advanced computing programme for optimization and then robust, low uncertainty predictions of the plasma, and materials performance; and (viii) comprehensive *in silico* design, digital prototypes and finally models of components and systems to support convincing qualification of the solutions. Solutions to the last challenge, in particular, can have much wider application to large-scale industrial activities where large steps can reduce development time and cost.

## 3. Overview of UKAEA's contributions to the fusion researchand development challenges

The breadth and depth of experience and wide knowledge of the integrated fusion needs accumulated by UKAEA over the years, together with the design, construction and operation of major fusion facilities, has led to an organization capable of both specialist and integration contributions. UKAEA has developed a portfolio of facilities and capabilities which allow us, in partnership with EUROfusion,^2^ other national laboratories around the world, academia and industry, to address the challenges identified in §2. A brief overview of these capabilities and recent examples of achievements are outlined below, delineated against each of the major challenges on the path to producing fusion electricity. The modelling and design challenges (vii) and (viii) are imbedded in the other six.

The main UKAEA facilities existing and in development are:

— JET [1], the world's largest and most capable fusion facility today, capable of using tritium, is operated by the UK for exploitation as a key part of the EUROfusion roadmap to fusion electricity (https://www.euro-fusion.org/eurofusion/roadmap/). JET is a model for ITER [2] and is the best facility to mitigate risks ahead of ITER operation.
— MAST Upgrade [4,8], together with NSTX-U [9], are the world's largest ‘spherical’ tokamaks. MAST Upgrade has unique features focused on the exhaust issue, challenge (ii), and will be developed and exploited with EUROfusion. MAST Upgrade will have a very extensive set of detailed measurements using, for example, advanced spectroscopy, atomic physics, lasers, neutrons and microwaves [5].
— The UKAEA Materials Research Facility (MRF) (http://www.ccfe.ac.uk/mrf.aspx), allows advanced analysis and tests of materials relevant to fusion (and fission) (http://www.ccfe.ac.uk/assets/Documents/Other/MRF_Brochure_mediumresolution.pdf), including materials activated and damaged after neutron irradiation or exposed to tritium. It is a part of the National Nuclear User Facilities (NNUF) (http://www.nnuf.ac.uk/), strongly linked to the Henry Royce Institute (http://www.royce.ac.uk/).
— Remote Applications in Challenging Environments (RACE) (http://www.race.ukaea.uk/), is a new centre to develop remote maintenance and robotics techniques for fusion and other applications. It builds on the extensive experience of remote handling on JET, and the EUROfusion DEMO remote maintenance programme is led from RACE.
— The Hydrogen-3 (tritium) Advanced Technology (H3AT) facility (https://www.gov.uk/government/news/86-million-boost-for-uk-nuclear-fusion-programme) is a new facility planned to be opened in 2021. It will have facilities for exposing materials to tritium, developing efficient tritium separation and purification techniques, and R&D on tritium removal at low and high concentration from solid, liquid and gaseous materials.
— A new Fusion Technologies Facility (FTF) (http://www.nnuf.ac.uk/) is planned to open in 2021, and will include an evolving range of bespoke capabilities, in three sub-facilities: a Materials Technology Laboratory focusing on small sample testing techniques, a Joining and Advanced Manufacturing Technology Laboratory (JAMTL) and the Module Testing Facility to offer a first step in fusion-relevant testing environments for metre-scale components, e.g. combined thermomechanical, hydromechanical tests and static and dynamic magnetic fields.

**Figure 2.**
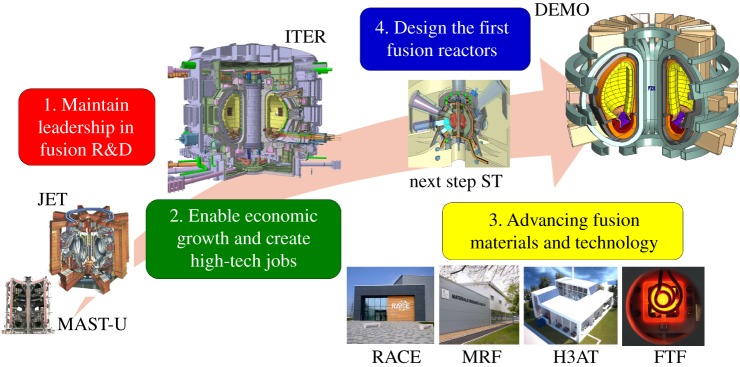
The roadmap to delivering fusion power, reflecting the goals of the UK Atomic Energy Authority. (Online version in colour.)

These facilities are part of a roadmap to the first demonstration reactor to produce fusion power (DEMO). Figure 2 shows this roadmap from present-day devices, JET and MAST Upgrade, through the first burning plasmas in ITER, to designing the first reactors, DEMO, and exploring the spherical tokamak as a possible way to drive down the cost of fusion power.

### (a) Creation and sustainment of a controlled burning plasma

ITER is the flagship facility on the European roadmap to fusion energy and JET plays a critical role in the development of integrated plasma scenarios of operation which are needed for ITER and for reactors thereafter. ITER's primary goal is to demonstrate a power gain of 10 in the plasma (*Q *= 10). DEMO and power plants would aim for higher *Q*, but in general ignition (*Q* infinite) is not sought; rather, the aim is a controlled burn where the plasma is mainly heated by the fusion alpha-particles, augmented by a modest amount of auxiliary heating to allow the fusion power to be more accurately controlled.

Predictions of plasma performance in ITER are mainly based on models developed from a large database of tokamak results in deuterium plasmas, studied in devices with carbon plasma-facing components and externally supplied heating, but these cannot yet capture all aspects of the conditions anticipated in ITER, e.g. ITER's mixture of high and low Z wall materials (tungsten and beryllium) change the boundary conditions on the core plasma; the transport of heat and particles changes with the fuel isotope (i.e. D and T); alpha-particle heating is determined nonlinearly by the temperature and pressure profiles; fast alpha-particles can, on the one hand, excite plasma instabilities (in particular Alfvén eigenmodes) and, on the other hand, can reduce turbulent transport.

JET is the world's reference facility to prepare for ITER operation, with several unique aspects, including being the only machine of its size with high-performance, high energy plasmas, the only machine able to operate with tritium fuel and the only machine with an ITER-like mixture of wall materials, namely beryllium and tungsten (chosen to reduce tritium retention in the vessel walls—an important operational constraint). A major task is to mitigate the main risks facing ITER in its research programme, so JET is focused on developing ITER-relevant integrated plasma scenarios with both deuterium and tritium (the isotope dependence of performance is a key scientific question), developing techniques to moderate the effects of losing control of the plasma (disruptions), and a range of other topics. The transfer of the results and experience to ITER plasmas will make extensive use of progressively improved modelling (the mixture of physics mechanisms will change somewhat when moving from JET to ITER—purely empirical extrapolation is not sufficient).

DEMO devices are likely to need advances compared with the reference ITER plasmas, for example high radiative losses from seeded impurities to spread the heat load on the plasma-facing components more widely. If steady state is needed in a power plant, this will require further developments; non-inductive current drive needed for steady state can require large amounts of power, reducing the overall efficiency of a power plant. The new EU-Japan tokamak JT-60SA (http://www.jt60sa.org/pdfs/JT-60SA_Res_Plan.pdf) will have a strong focus on long pulse and steady state, and UKAEA is engaged in modelling its plasma scenarios.

High-quality plasma modelling tools will underpin the design of high-performance plasmas on ITER and DEMO. JET will play an important role in their development. State-of-the-art models must describe: turbulent transport of heat and particles in the core and edge plasma; stability; fast particle physics; heating and current drive physics; strong radiative cooling by seeded impurities as a part of the exhaust solution; the exhaust plasma in the scrape-off layer and divertor (outside the region of nested magnetic surfaces); and plasma wall interactions, and other aspects of plasma dynamics.

Transport and stability set the minimum size of a plasma which can generate net power—scaling from the best substantiated operating scenarios lead to the dimension chosen for ITER, and since DEMO will have to generate substantially more power to generate net electricity, it needs to be somewhat larger, if based on the same plasma regimes. A deeper understanding of the transport processes that will occur in burning plasmas may allow alternative scenarios to be found which could allow more compact plasmas, including smaller major radius (i.e. lower aspect ratio, as explored on MAST Upgrade). Today, these are far from the maturity needed to be considered for DEMO and power plants, although several mechanisms for turbulence reduction have been seen in experiment and theory [10,11]. Some of the ideas being developed, including by other institutes including UK universities (for example, van Wyk *et al.* [12]), can be explored with MAST Upgrade.

#### (i) Recent results

Interestingly, the ITER-like metal wall on JET introduced operational constraints that initially resulted in reduced plasma performance compared to the previous carbon wall [13,14]. However, a combination of a divertor heat-handling technique together with central heating to expel impurities from the core enabled the performance in JET to be restored to previous levels [15,16]. Results from the first ITER-like wall campaign have shown a significant (approx. 20×) decrease in fuel retention compared with the previous carbon wall [17], and dust/particulate generation in the divertor is a factor approximately 100× lower [18].

### (b) Controlling the exhaust of heat and helium ‘ash’

Present tokamaks usually operate with a modest fraction of power radiated from the main plasma, to avoid performance degradation. This means that most of the exhaust power is channelled along field lines to the divertor targets. In burning plasmas, this leads to very high power densities on the materials, and in ITER and particularly DEMO, this can easily exceed the material limits [19]. Furthermore, these limits are expected to be reduced after neutron irradiation, which degrades the materials' mechanical and heat conduction properties (https://www.gov.uk/government/organisations/uk-atomic-energy-authority). If the plasma at the material surface is too hot, sputtering of the material generates impurities which can degrade the core plasma, and in particular, erosion of the surface can lead to unacceptably short lifetimes or drive designs with worse thermal performance. To address this problem, various strategies are adopted: increased radiative losses from the main plasma to spread the load over the first wall [20], spreading of the heat load on the divertor by modifying magnetic geometry [21], and most importantly making ‘detached’ plasmas where the plasma is cooled by injecting deuterium and radiating impurities into the divertor [22]. A detached plasma can have very low power flux at the materials (but often large particle fluxes); the challenge is to ensure that it remains securely detached, without the detachment region and seed impurities degrading the core plasma, or even causing a disruption. The core plasma scenario may need to be adapted in other ways, for example, fast transients (such as edge localized modes, a type of plasma instability that are almost ubiquitous in ITER-like plasma scenarios) can severely shorten the lifetime of the plasma-facing components and can break through the detached region. Slow transients, such as variations in the fusion power, can also cause reattachment.

MAST Upgrade is a uniquely flexible facility for studying the underlying physics of plasma exhaust and comparing different geometries for exhausting heat and particles from hot plasmas. It is particularly notable in its capability to operate with a super-X configuration optimized for fully exploring the characteristics of this divertor concept (http://www.ccfe.ac.uk/mast_upgrade_project.aspx, http://www.ccfe.ac.uk/assets/documents/other/MAST-U_RP_v4.0.pdf). The super-X has features well suited for spherical tokamaks and provides a bounding configuration for conventional aspect ratio devices such as the EU DEMO. It is important to emphasize that MAST Upgrade is primarily intended to be a very flexible test-bed to study exhaust physics in many divertor configurations, from conventional divertor (as in JET and ITER) to X-divertor, snowflake, super-X and even extended inner-leg configurations, in single and double null versions [23]. The design of exhaust solutions for DEMO will rely heavily on theory-based models, given the change in physics parameters from existing or planned devices, so MAST Upgrade will be used to confront and thus improve the models that allow the next steps to be taken, rather than being a prototype [7].

As one example of the new physics MAST Upgrade can address, we consider the control of plasma detachment: the extent of the radiative and detachment regions, their expansion/movement from the target towards the X-point region and hot core plasma are not well understood. They involve a detailed interaction of the atomic physics of ions and neutrals, and the local and non-local turbulent and classical transport of Maxwellian and non-Maxwellian electrons and multiple ion species. These studies will be supported by flexible plasma heating, fuelling and pumping to explore the parameter space and to act as control actuators, and a wide range of high-resolution diagnostics to explore the physics, test models in detail and provide advanced control ‘observers’.

#### (i) Recent results

Observations of plasma filaments in the scrape-off layer (SOL) and divertor and new modelling are starting to reveal what sets the filament behaviour and its relation to the density SOL width [24–26]. Finding a way to change their behaviour to increase the width could provide a means of alleviating the exhaust problem. Modelling of detachment shows how the variation in magnetic field strength along the divertor can play a key role in detachment control and the operation window [21,27].

### (c) Developing materials for fusion reactors

The focus of the UKAEA programme is on structural and plasma-facing materials for DEMO [28], which are low activation ferritic steels [29], such as EUROFER, and tungsten (or alloys), respectively. Critical issues for the steels include the operating temperature range after irradiation, especially the ductile-to-brittle transition temperature [30] (a lower bound to operation under strain), which increases with irradiation, especially by 14 MeV neutrons which generate helium in the material, driving embrittlement, and phase changes which reduce the strength (setting the upper temperature limit). Widening the operating window at both ends is important to increase the lifetime and improve the thermodynamic efficiency of the fusion plant by allowing higher temperature operation [31].

At present, there is no high flux, high fluence source of a fusion-like spectrum of neutrons (this is the purpose of the proposed IFMIF [32,33] and IFMIF/DONES [34] facilities), so predictions and materials selection and development have to use advanced multiscale modelling, which is extremely challenging theoretically and computationally. However, much of the material in DEMO will be subjected to a fission-like neutron spectrum, so progress can be made there with materials test reactors and advanced analysis of the irradiated samples, developing theory and modelling interactively. Furthermore, ion irradiation can sometimes be used as a partial proxy for neutrons, when accompanied by suitable theory, and this allows accelerated collection of data. A major challenge is developing high-fidelity multiscale modelling, bridging from atomistic to macroscopic properties—engineering design needs the macroscopic properties of materials, including failure modes, of materials after irradiation. The properties of materials are also critically dependent on the manufacturing process, and since additive manufacture provides a highly promising path for affordable fabrication of complex structures (such as optimized heat transfer with very small coolant complex-path channels), the properties of these materials need to be predicted and tested.

The UK universities and research organizations such as UKAEA work together at the forefront of advancing the *ab initio* understanding of radiation damage (including gas embrittlement) of fusion steels and tungsten, with exacting comparisons with experiments, now complemented by analysis facilities in the MRF. The MRF's role is to allow fission and fusion scientists to process and analyse samples too radioactive for university premises but not requiring the facilities of a nuclear licenced site. The MRF has a purpose-designed building, hot cells, processing equipment and a range of fine-scale mechanical, thermo-physical and electron microscopy characterization equipment. It will expand significantly in the coming years, with the emphasis on further mechanical and thermo-physical testing capability over a wide range of scales and temperatures, plus further hot cell capability, allowing larger scale testing. The MRF will be exploited to examine how radiation degrades materials and to analyse JET tungsten and beryllium tiles after exposure to deuterium and tritium, and often high heat and particle flux. Moving to larger scales, studies of the engineering properties will require the Materials Technology Laboratory (see below), which will operate in harmony with the MRF, leading to the design rules needed for components and bringing in industry expertise.

#### (i) Recent result

A spin-lattice dynamics simulation program, Spilady [35], has been developed and made widely available. This is an important tool for modelling the critical effects of magnetism in fusion steels. A new scaling for the size distribution of defects in irradiated tungsten has been discovered, in work carried out in collaboration with co-workers from Finland, Oxford University and Argonne National Laboratory [36,37].

### (d) Developing components to work inside the tokamak

Components, such as divertor targets and other plasma-facing components which combine tungsten, CuCrZr or steel-cooling pipes and steel structures, need to operate with high reliability. Steel structures such as the blanket modules will have many welds, some of which will need to be cut and re-welded during maintenance. The performance and, in particular, the failure modes (hence lifetime) of many components are very challenging to predict since they combine the properties of the base materials, joints between dissimilar materials and modified regions such as welds.

The approach to these challenges is phased. Initially joints and components will be developed with the combined loads without including irradiation effects, and in any case, the components need to work well before they are significantly irradiated. It is assumed that the best components will use the radiation-resilient materials identified or developed by the fusion community's materials science programmes and manufactured taking account, as far as possible, of expected effects of irradiation on, e.g. joints and welds. In accordance with this thesis, the new FTF is the UKAEA's vehicle. These bespoke facilities tailored to meet the needs of fusion will enable thermal, mechanical, hydraulic and electromagnetic tests on prototype components under the conditions experienced inside fusion reactors (without the nuclear effects at this stage). Comprised of three independent laboratories, the FTF offers a complete development life cycle for materials and components. The Materials Technology Laboratory (MTL) specializes in the development and qualification of small sample testing techniques to reduce costs and volumes of testing and offering ‘in-service’ examination. Exploiting the opportunity to bring cutting edge advances in testing techniques into new nuclear design codes, the MTL will develop multi-axial testing, fracture mechanics of brittle materials and true stress true strain analysis among other techniques. The MTL contains two load frames of 5 and 7.5 kN capable of operating at 700°C and 1000°C, respectively (so can reach the upper limits expected for steel operation), with operation in inert atmospheres and *in vacuo* with Digital Image Correlation measurement. Automated hardness testing, sample preparation and heat treatment and characterization capabilities are also provided.

The JAMTL will enable the development of critical material joining and manufacturing technologies required to deliver fusion, such as the qualification of laser welding, developed in the EUROfusion remote maintenance project. Building on previous experience, powder and wire metallurgical advanced manufacturing (AM) methods are being developed to create novel cooling architectures in plasma-facing components such as divertors and functionally graded joints, supporting the development of a high-quality industrial supply chain for these applications. The development of fusion compatible non-destructive testing techniques and condition-monitoring sensors, in addition to manufacturing for maintenance, are key themes for JAMTL. These activities are supported by a test stand for small component testing (heat by induction to verify extremes (HIVE)), to provide fusion-relevant heat transfer that allows rapid prototyping of AM and sensor technologies [38].

The third member of the FTF is the Module Test Facility (MTF) that is planned to offer fusion-relevant testing environments for metre-scale components in static and time-varying magnetic fields to investigate the impact of induced forces and plasma disruptions [39]. A key feature of the MTF will be the high degree of instrumentation and data collection and handling capability that will allow the adoption of virtual twin philosophy for component engineering. This allows the validation of computer models of the component that may be used for lifetime studies and off normal event simulations, avoiding expensive prototype build.

As well as developing techniques for manufacturing effective components, these facilities, and the MRF, will help provide lifetime estimates and failure modes, important both for component optimization and for determining the maintenance approach to maximize plant availability.

#### (i) Recent result

Advanced component analysis with image-based finite-element modelling has been used to determine and correct manufacturing flaws leading to better high heat flux components [40]. Additive manufacturing has been used to make high heat flux prototypes with narrow and optimized internal cooling channels that would not have been possible to make by conventional approaches [39].

### (e) Achieving a high availability for the fusion plant

As indicated above, a fusion plant comprises a wide range of science and technology, with many interactions and constraints, and DEMO(s) will provide the first tests. Achieving a consistent design from plasma to electricity grid is itself a major challenge. However, it is essential that the plant operates predictably and reliably (so each component and system needs to have extremely high reliability since there are so many), and that maintenance can be done rapidly minimizing down-time and, in a timely way, pre-empting component failures as far as possible. All maintenance of the tokamak structure during operation with DT plasmas (and high-performance DD plasmas) will need to be done remotely [41]. Therefore, remote maintenance needs to be designed in from the outset, and it has a major impact on design and architecture choices [42,43]. UKAEA has chosen to put a major focus on remote maintenance since it is so key to the holistic plant design [44].

A fusion reactor is perhaps the ultimate challenging environment for reliable operation and maintenance [21]: ∼500 K, vacuum, liquid metals, confined spaces and kGy/hour radiation. Remote maintenance (RM) will be a fusion power plant ‘device defining driver’ whether a power plant is on a similar scale to ITER or a way has been found to make a small modular reactor based on a spherical tokamak. RM needs to take into account the design, build, inspection, maintenance, operation and decommissioning of the power plant (and vice versa as above). For a fusion power plant, necessary RM components must be developed to the appropriate technology readiness level to demonstrate viability before the next (more expensive) phase of design and/or build. As well as using virtual engineering, mock-ups will need to be designed and fabricated, then qualified to enable regulation of power plants.

To produce a robust and qualified remote maintenance system requires: augmented and virtual reality testing; advanced control systems for a neutron environment; cutting and joining radiation-damaged steel pipes and inspecting to ensure acceptable quality; manipulating large irradiated components to extremely tight tolerances, all to nuclear standards. The key technical risks for the RM of power plants centre around the movement of large ‘flexible’ loads such as groups of blanket modules which may together have a mass approaching 100 t, and rapid and reliable connection of many component service pipes to satisfy the requirements of a nuclear regulator.

RACE provides a flexible facility which spans the development cycle for robotic maintenance solutions, from the *in silico* virtual design, to prototype testing, and operation of the JET remote maintenance system for two decades. The UK plays a substantial role in the design of remote maintenance systems for both ITER (where UK industry is involved in all contracts issued so far) and DEMO. There are strong synergies with other areas where human access is undesirable or impossible—for example, the target area of the European Spallation Source where UKAEA is working on the remote maintenance.

#### (i) Recent result

Following the theme of holistic design, a first view of the integrated remote maintenance of an EU DEMO has been created [45]. The handling of massive blanket modules requires innovative approaches (the ‘crane’ cannot be many times the mass of the blanket module—i.e. unlike the approach used for most crane systems), and a hybrid kinematic manipulator concept has been developed building on approaches used in other industries [44].

### (f) Breeding and managing tritium

Fusion plants must breed all their tritium, with some margin to cover decay during maintenance periods, tritium temporarily resident in materials and the tritium plant and not available for fuelling the plasma, and for starting up new fusion plants [46]. In addition, the site inventory will be tightly restricted by the regulator [47], so the amount of tritium outside the plasma at any time must be minimized and losses eliminated wherever possible. This means that very efficient low inventory fuelling systems are needed, the volume of the tritium plant must be minimized, there needs to be fast extraction of tritium from the breeding material and the amount of tritium retained in materials has to be minimized. Finally, the tritium inventory of items leaving the plant site must be kept to extremely low levels to simplify waste handling and minimize its cost [48]. The breeding occurs in the blanket surrounding the plasma which also converts the energy in the fusion neutron into bulk thermal energy, which is the primary heat source for the electricity-generating turbines and, at the same time, shields the vacuum vessel so it can be made from more conventional steels; the blanket is an interesting multi-disciplinary project in itself.

H3AT will offer the ability to pursue tritium-related R&D in several key areas that currently challenge fusion. Detritiation is one example relevant to ITER and DEMOs and is required at various points in the lifecycle [49]. In the fuel cycle, isotope separation and rebalancing is critical, particularly as the process time is a major contributor to the tritium inventory required to start a fusion power plant. H3AT will provide facilities to support studies in these areas together with tritium pumping and storage technologies. Recovering tritium from coolants and materials will be essential to minimize the active waste inventory of ITER and DEMO, and H3AT will provide facilities for R&D on tritium removal at low and high concentration from solid, liquid and gaseous materials.

Preventing or minimizing tritium migration is obviously required along with the development of tritium removal techniques from different breeder blanket designs, and H3AT will offer facilities to investigate these areas. Tritium control, monitoring and accountancy are all essential for operation and licensing, and R&D programmes for technologies in these areas can be accommodated.

#### (i) Recent result

The idea of a small inner loop in the DEMO fuel cycle is likely to be key to an acceptably low tritium inventory. The concept [50] originated at the Karlsruhe Institute of Technology, and UKAEA has been collaborating with its further development [51].

### (g) Developing an integrated design for fusion reactors

As stressed above, an integrated approach from the outset is critical for holistic fusion reactor design. The main systems and features to integrate in an MCF reactor are

— the plasma;
— superconducting magnets and their high strength support structures;
— blanket (converts fast neutrons to heat and tritium and shields the vessel);
— divertor (for exhaust);
— heating and current drive systems for plasma production, sustainment and control;
— measurement systems (plasma and plant);
— tritium plant and fuelling system;
— balance of plant—the turbines, power conversion systems, cryoplant, power supplies;
— safety and waste; and
— qualification process to satisfy regulators and investors.

Major strides in integration have been taken in recent years, especially in ITER and EUROfusion's DEMO design activity. There are many examples of unexpected issues emerging when integration is attempted. For instance, the number of toroidal field coils affects the viability of remote maintenance; first wall armour to protect from plasma heating can reduce the tritium breeding; the blanket operating temperature (thermodynamic efficiency) is constrained by steel properties and the coolant pumping power, and, in turn, constrains the fusion power from the plasma; short pulse length substantially reduces the recirculating power to sustain the plasma and may increase the overall efficiency compared with steady state.

Realizing DEMO and the First-Of-A-Kind Fusion Power Plants requires a multi-disciplinary approach with the capabilities and facilities to address all of the challenges outlined in §2 simultaneously. This integrated design capability will need to bring together a top-level fusion power plant design capability, incorporating systems codes with cutting edge models for all aspects of the design, socio-economic assessments, commissioning, maintenance, operations, waste management and decommissioning, with a rapid prototyping and validation programme. The prototypes, and later DEMO, can be used as test beds to subject the virtual models to representative and extreme scenarios to understand real world performance, failure modes and through-life issues. UKAEA will work with a wide range of partners in industry and academia, both nationally and internationally, to move along this path to delivering fusion reactor designs.

A factor not discussed much so far is the cost, but this will be critical in the end: the overall cost of electricity and also the capital cost of the plant, including the largest single investment, the tokamak itself. The holistic approach described in this paper applies to any concept, and it allows coherent exploration of alternative approaches which might lead to lower cost of the tokamak core, by uncovering or stimulating plasma and technology innovations that are not applicable to the ITER-like approach. To this end, UKAEA will, alongside its major contributions to the EU DEMO programme, work with collaborators to seek innovations and features that would allow smaller physical size and lower capital cost, focusing on the spherical tokamak and making a key next step with MAST Upgrade, but always taking a holistic view.

Finally, it is also worth noting that successful delivery of fusion power will be dependent on a supply of highly trained capable scientists and engineers. UKAEA has a strong training programme at all levels, from apprentices, to graduates, to post-graduate students and post-doctoral researchers.

## 4. Conclusion and future perspectives

Since 16 MW of fusion power was achieved in JET in 1997, the headline progress in fusion has appeared to the outside world to slow down. This belies substantial technical progress and greatly improved understanding of the science and technology in the field. However, it is representative of the fact that fusion requires a burning plasma, where the fusion reaction provides products which sustain the reaction, before fusion on a commercial basis can be considered possible. ITER will provide that demonstration, and as such is critical to the success or failure of commercial fusion power. UKAEA will continue to contribute significantly to ensure ITER reaches its goals, in many technical and scientific areas and in providing expert advice to industry—many of ITER's needs can be satisfied by existing industrial capabilities.

However, while ITER will show fusion is possible, it will not provide net fusion electricity. UKAEA's evolving portfolio of facilities will help to address some of the challenges in the transition to DEMO and power plants, but a concerted, multinational endeavour will be needed in parallel with ITER to address them all appropriately. For instance, the MRF is an important facility for testing and validating numerical models of small material samples irradiated by low-energy (usually fission-spectrum) sources. However, only with samples exposed to a fusion-relevant spectrum of very energetic neutrons at high fluence can materials be qualified for use in internal reactor components really be validated, and this requires a major facility such as IFMIF/DONES, which is envisaged as a multinational collaboration.

As well as a multinational collaboration to provide the requisite capability and facilities to enable fusion to be commercialized, a supply chain capable of designing and building fusion reactors must also be developed. UKAEA plays a central role in enabling UK industry to deliver fusion-specific components and systems for ITER and will increasingly foster contributions from industrial partners and transfer knowledge to address the fusion challenges to the supply chain.

The realization of fusion remains elusive, but its potential remains vast. ITER will be the first burning plasma and a DEMO designed on the same basic principles as ITER, while incorporating discoveries and innovations as far as possible, is the highest confidence path. Given the impact cost-competitive and reliable fusion power would have in meeting the world's demands for reliable low carbon energy, it is important to keep innovating and optimizing at all levels from materials to whole concepts (e.g. exploring spherical tokamaks) to bring down both the capital and the operating cost. Ultimately, the penetration of fusion power into the market may be driven by capital cost of reactor build more than the overall cost of electricity.

## Footnotes

1 Other magnetic confinement systems are also pursued, the next leading one being the stellarator, with Wendelstein 7-X (https://www.ipp.mpg.de/w7x) and the Large Helical Device (https://www-lhd.nifs.ac.jp/pub/index_e.html) being the major facilities, in Germany and Japan, respectively. Alternative schemes are pursued too, most notably inertial fusion where small pellets of D–T fuel are compressed and heated, usually by high power lasers, the largest experiment of this type being NIF in the USA (https://lasers.llnl.gov/).

2 EUROfusion, https://www.euro-fusion.org/, is a consortium of European fusion laboratories formed to conduct a co-ordinated R&D programme.

## References

[1] Litaudon X et al. 2017 Overview of the JET results in support to ITER. Nucl. Fusion 57, 102001 (10.1088/1741-4326/aa5e28)

[2] Shimada M et al. 2007 Progress in the ITER physics basis. Nucl. Fusion 47, S1 (10.1088/0029-5515/47/6/S01)

[3] Keilhacker M et al. 1999 High fusion performance from deuterium-tritium plasmas in JET. Nucl. Fusion 39, 209 (10.1088/0029-5515/39/2/306)

[4] Watkins ML et al. 1999 Physics of high performance JET plasmas in DT. Nucl. Fusion 39, 1227 (10.1088/0029-5515/39/9Y/302)

[5] Federici G et al. 2014 Overview of EU DEMO design and R&D activities. Fusion Eng. Design 89, 882 (10.1016/j.fusengdes.2014.01.070)

[6] Federici G, Biel W, Gilbert MR, Kemp R, Taylor N, Wenninger R 2017 European DEMO design strategy and consequences for materials. Nucl. Fusion 57, 092002 (10.1088/1741-4326/57/9/092002)

[7] Morris AW, Kirk A, Lipschultz B, Militello F, Moulton D, Walkden NR 2018 MAST upgrade divertor facility: a test bed for novel divertor solutions. IEEE Trans. Plasma Sci. 46, 1217–1226. (10.1109/TPS.2018.2815283)

[8] Kirk A et al. 2017 Overview of recent physics results from MAST. Nucl. Fusion 57, 102007 (10.1088/1741-4326/aa65e0)

[9] Menard JE et al. 2017 Overview of NSTX upgrade initial results and modelling highlights. Nucl. Fusion 57, 102006 (10.1088/1741-4326/aa600a)

[10] Valovic M et al. 2009 Scaling of H-mode energy confinement from *I*_p_ and *B*_T_ in the MAST spherical tokamak. Nucl. Fusion 49, 075016 (10.1088/0029-5515/49/7/075016)

[11] Field AR et al. 2011 Plasma rotation and transport in MAST spherical tokamak. Nucl. Fusion 51, 063006 (10.1088/0029-5515/51/6/063006)

[12] van Wyk F, Highcock EG, Schekochihin AA, Roach CM, Field AR, Dorland W 2016 Transition to subcritical turbulence in a tokamak plasma. J. Plasma Phys. 82, 905820609 (10.1017/S0022377816001148)

[13] Beurskens MNA et al. 2014 Global and pedestal confinement in JET with a Be/W metallic wall. Nucl. Fusion 54, 043001 (10.1088/0029-5515/54/4/043001)

[14] Maggi CF et al. 2015 Pedestal confinement and stability in JET-ILW ELMy H-modes. Nucl. Fusion 55, 113031 (10.1088/0029-5515/55/11/113031)

[15] Challis CD et al. 2015 Improved confinement in JET high-plasmas with an ITER-like wall. Nucl. Fusion 55, 053031 (10.1088/0029-5515/55/5/053031)

[16] Kim H-T et al. 2018 High fusion performance at high *T_i_*/*T_e_* in JET-ILW baseline plasmas with high NBI heating power and low gas puffing. Nucl. Fusion 58, 036020 (10.1088/1741-4326/aaa582)

[17] Brezinsek S et al. 2013 Fuel retention studies with the ITER-Like Wall in JET. Nucl. Fusion 53, 083023 (10.1088/0029-5515/53/8/083023)

[18] Baron-Wiechec A et al. 2015 First dust study in JET with the ITER-like wall; sampling, analysis and classification. Nucl. Fusion 55, 113033 (10.1088/0029-5515/55/11/113033)

[19] You JH et al. 2016 European DEMO divertor target: operational requirements and material-design interface. J. Nucl Mater. Energ. 9, 171 (10.1016/j.nme.2016.02.005)

[20] Wenninger R et al. 2015 DEMO exhaust challenges beyond ITER. In *Proc. 42nd EPS Conference on Plasma Physics 2015, Lisbon, Portugal, 22--26 June*, Paper P4.110. Mulhouse, France: EPS.

[21] Lipschultz B et al. 2016 Sensitivity of detachment extent to magnetic configuration and external parameters. Nucl. Fusion 56, 056007 (10.1088/0029-5515/56/5/056007)

[22] Matthews GF 1995 Plasma detachment from divertor targets and limiters. J. Nucl. Mater. 220, 104 (10.1016/0022-3115(94)00450-1)

[23] Harrison JR et al. 2016 Enhancements to MAST Upgrade to address the EUROfusion Plasma Exhaust Strategy. In *Proc. 43rd EPS Conference on Plasma Physics, 2016, Leuven, Belgium, 4--8 July*. Mulhouse, France: EPS.

[24] Harrison JR et al. 2015 The appearance and propagation of filaments in the private flux region in mega amp spherical tokamak. Phys. Plasmas 22, 092508 (10.1063/1.4929924)

[25] Walkden NR, Harrison J, Silburn SA, Farley T, Henderson SS, Kirk A, Militello F, Thornton A 2017 Quiescence near the X-point of MAST measured by high speed visible imaging. Nucl. Fusion 57, 126028 (10.1088/1741-4326/aa8512)

[26] Militello F, Omotani JT 2016 Scrape off layer profiles interpreted with filament dynamics. Nucl. Fusion 56, 104004 (10.1088/0029-5515/56/10/104004)

[27] Moulton D, Harrison J, Lipschultz B, Coster D 2017 Using SOLPS to confirm the importance of parallel area expansion in Super-X divertors. Plasma Phys. Control. Fusion 59, 065011 (10.1088/1361-6587/aa6b13)

[28] Stork D et al. 2014 Developing structural, high-heat flux and plasma facing materials for a near-term DEMO fusion power plant: the EU assessment. J. Nucl. Mater. 455, 277 (10.1016/j.jnucmat.2014.06.014)

[29] Gelles DS 1996 Microstructural examination of commercial ferritic alloys at 200 dpa. J. Nucl. Mater. 233–237, 293–298. (10.1016/S0022-3115(96)00222-X)

[30] Gaganidze E, Schneider H-C, Dafferner B, Aktaa J 2007 Embrittlement behavior of neutron irradiated RAFM steels. J. Nucl. Mater. 367, 81–85. (10.1016/j.jnucmat.2007.03.163)

[31] Zinkle SJ 2005 Fusion materials Science: Overview of challenges and recent progress. Phys. Plasmas 12, 058101 (10.1063/1.1880013)

[32] Ibarra A et al. 2007 Recent EU activities for IFMIF EVEDA in the framework of the broader approach. Fus. Eng. Design 82, 2422–2429. (10.1016/j.fusengdes.2007.07.029)

[33] Knaster J et al. 2017 Overview of the IFMIF/EVEDA project. Nucl. Fusion 57, 102016 (10.1088/1741-4326/aa6a6a)

[34] Ibarra A, Heidinger R, Barabaschi P, Mota F, Mosnier A, Cara P, Nitti FS 2014 A stepped approach from IFMIF/EVEDA toward IFMIF. Fusion Sci. Technol. 66, 252–259. (10.13182/FST13-778)

[35] Ma P-W, Dudarev S SPILADY: a spin-lattice dynamics simulation program. See http://www.ccfe.ac.uk/spilady_code.aspx.

[36] Sand E et al. 2013 High-energy collision cascades in tungsten: dislocation loops structure and clustering scaling laws. EPL 103, 46003 (10.1209/0295-5075/103/46003)

[37] Mason DR, Sand AE, Yi X, Dudarev SL 2018 Direct observation of the spatial distribution of primary cascade damage in tungsten. Acta Mater. 144, 905 (10.1016/j.actamat.2017.10.031)

[38] Hancock D et al. 2018 Exploring complex high heat flux geometries for fusion applications enabled by additive manufacturing. Fusion Eng. Design 136, 454–460.

[39] Sugihara M et al. 2007 Disruption scenarios, their mitigation and operation window in ITER. Nucl. Fusion 47, 337 (10.1088/0029-5515/47/4/012)

[40] Barrett TR et al. 2016 Progress in the engineering design and assessment of the EuropeanDEMO first wall and divertor plasma facing components. Fusion Eng. Design 109–111, 917 (10.1016/j.fusengdes.2016.01.052)

[41] Buckingham R, Loving A 2016 Remote-handling challenges in fusion research and beyond. Nat. Phys. 12, 391–393. (10.1038/nphys3755)

[42] Keep J, Wood S, Gupta N, Coleman M, Loving A 2017 Remote handling of DEMO breeder blanket segments: blanket transporter conceptual studies. Fusion Eng. Design 124, 420 (10.1016/j.fusengdes.2017.02.016)

[43] Skilton R et al. 2018 MASCOT 6: Achieving high dexterity tele-manipulation with a modern architectural design for fusion remote maintenance. Fus. Eng. Design 136, 575–578.

[44] Agudo V et al. 2017 International Symposium on Fusion Nuclear Technology, P3–165, in press.

[45] Crofts O et al. 2016 Overview of progress on the European DEMO remote maintenance strategy. Fusion Eng. Design 109–111, 1392 (10.1016/j.fusengdes.2015.12.013)

[46] Kovari M, Coleman M, Cristescu I, Smith R 2018 Tritium resources available for fusion reactors. Nucl. Fusion 58, 026010 (10.1088/1741-4326/aa9d25)

[47] Taylor N, Cortes P 2014 Lessons learnt from ITER safety and licensing for DEMO and future nuclear fusion facilities. Fusion Eng. Design 89, 1995 (10.1016/j.fusengdes.2013.12.030)

[48] Broden K, Edwards R, Lindberg M, Rocco P, Zucchetti M 1998 Waste from fusion reactor: a comparison with other energy producing systems. Fusion Eng. Design 42, 1 (10.1016/S0920-3796(97)00150-6)

[49] Bekris N, Caldwell-Nichols C, Doerr L, Glugla M, Penzhorn R-D, Ziegler H 2002 Possible techniques for the detritiation of first wall materials from fusion machines. J. Nucl. Mater. 307, 1649 (10.1016/S0022-3115(02)01131-5)

[50] Giegerich T, Day C 2014 The KALPUREX-process – a new vacuum pumping process for exhaust gases in fusion power plants. Fusion Eng. Design 89, 1476 (10.1016/j.fusengdes.2014.03.082)

[51] Day C, Butler B, Giegerich T, Lang PT, Lawless R, Meszaros B 2016 Consequences of the technology survey and gap analysis on the EUDEMO R&D programme in tritium, matter injection and vacuum. Fusion Eng. Design 109, 299 (10.1016/j.fusengdes.2016.03.008)

